# The Long-Suffering Middle-Class

**Published:** 1892-04-02

**Authors:** 


### Passing Topics.

### THE LONG-SUFFERING MIDDLE-CLASS.

A very interesting and instructive correspondence has been going on in the papers about the easy way in which that long-suffering section of our countrymen and countrywomen—the great middle-class—submits to numerous inflictions of various sorts conceived and imposed by the lawmakers in the interests of classes above and below them. We fear there is only too much truth in the assertion, that whether regarded as tenants or taxpayers, the members of the middle-class are awarded something more than their legitimate share of the burdens of citizenship, be they imperial, parochial, or domestic.

In one of the comedies of the first Lord Lytton, a gentleman constitutionally disposed to a lugubrious view of the situation, affirms that among the things an Englishman owes to his country are east winds, fogs, rheumatism, and taxes. If we are to accept Mr. Rose-Innes' doctrine, and he makes out a strong case, the most important part of the community may add wrong and outrage to their inheritance. They are, he insinuates, systematically and flagrantly plundered and put upon, while so much patience or so little spirit do they display, according to the view taken of their conduct, that they go on year by year without so much as a protest or a murmur, just as if they liked it.

It is in a similar spirit, we suppose, that a certain minute minority of the self-same class voluntarily assumes the burden in things charitable which ought to be shared by all. We will make Mr. Rose-Innes a present of this suggestion, so that he may string it with the other effective illustrations he has collected. We are not thinking only of public or institutional charity. Who is it supplies all those little local needs, which, in the aggregate, reach so heavy a yearly total in any given neighbourhood? Is there any proportional adjustment of the burden? On the contrary, do we not constantly witness actually or comparatively poor members of the "middle-classes" staggering under the weight of imposts, in these cases self-assumed, which, were they but equally distributed, might be more easily and more effectually borne?

The fact is that one class preys upon the other as to the manner born. There is in human nature, as in that of the inferior animals, a predatory instinct, and the people who appear the most free from its influence are those who are neither rich nor impecunious, whose life is not perhaps one long struggle with want, but who are yet so far removed from affluence that the indulgence of generosity calls for the self-sacrifice which adds zest to the exercise and stimulates the soul afresh. In no other way can we account for the indisposition to make use of their great opportunities which characterises ultra-rich people with but few exceptions.

Hence it is futile to urge that they miss a chief pleasure of life because to them there is no luxury in giving. Expostulation is like an embrocation applied to an organic disease. Rich people and our lawmakers are mostly of that order, or the scions of the rich, are far more impressed with the rights of property than with its duties and privileges, just as poor starving wretches are more sensible of their wrongs than of the best way of remedying them. As a consequence, whenever the "working classes" are clamorous, those just above them are made to provide the sop with which to stop their mouths. The unconscious selfishness of the rich is not more discouraging than the want of self-help in the poor. The intermediate classes are the victims of both.

Agricola.

